# Feasibility and Effects of Virtual Reality Motor-Cognitive Training in Community-Dwelling Older People With Cognitive Frailty: Pilot Randomized Controlled Trial

**DOI:** 10.2196/28400

**Published:** 2021-08-06

**Authors:** Rick Yiu Cho Kwan, Justina Yat Wa Liu, Kenneth Nai Kuen Fong, Jing Qin, Philip Kwok-Yuen Leung, Olive Suk Kan Sin, Pik Yuen Hon, Lydia W Suen, Man-Kei Tse, Claudia KY Lai

**Affiliations:** 1 Centre for Gerontological Nursing School of Nursing The Hong Kong Polytechnic University Hong Kong China (Hong Kong); 2 Department of Rehabilitation Sciences The Hong Kong Polytechnic University Hong Kong China (Hong Kong); 3 School of Nursing The Hong Kong Polytechnic University Hong Kong China (Hong Kong); 4 Mr. Kwok Hing Kwan Neighbour Elderly Centre Pok Oi Hospital Hong Kong China (Hong Kong)

**Keywords:** virtual reality, motor-cognitive training, cognitive frailty, game, feasibility, VR, training, older adults, frail, pilot study, randomized controlled trial

## Abstract

**Background:**

Cognitive frailty refers to the coexistence of physical frailty and cognitive impairment, and is associated with many adverse health outcomes. Although cognitive frailty is prevalent in older people, motor-cognitive training is effective at enhancing cognitive and physical function. We proposed a virtual reality (VR) simultaneous motor-cognitive training program, which allowed older people to perform daily activities in a virtual space mimicking real environments.

**Objective:**

We aimed to (1) explore the feasibility of offering VR simultaneous motor-cognitive training to older people with cognitive frailty and (2) compare its effects with an existing motor-cognitive training program in the community on the cognitive function and physical function of older people with cognitive frailty.

**Methods:**

A two-arm (1:1), assessor-blinded, parallel design, randomized controlled trial was employed. The eligibility criteria for participants were: (1) aged ≥60 years, (2) community dwelling, and (3) with cognitive frailty. Those in the intervention group received cognitive training (ie, cognitive games) and motor training (ie, cycling on an ergometer) simultaneously on a VR platform, mimicking the daily living activities of older people. Those in the control group received cognitive training (ie, cognitive games) on tablet computers and motor training (ie, cycling on the ergometer) sequentially on a non-VR platform. Both groups received a 30-minute session twice a week for 8 weeks. Feasibility was measured by adherence, adverse outcomes, and successful learning. The outcomes were cognitive function, physical frailty level, and walking speed.

**Results:**

Seventeen participants were recruited and randomized to either the control group (n=8) or intervention group (n=9). At baseline, the median age was 74.0 years (IQR 9.5) and the median Montreal Cognitive Assessment score was 20.0 (IQR 4.0). No significant between-group differences were found in baseline characteristics except in the number of chronic illnesses (*P*=.04). At postintervention, the intervention group (Z=–2.67, *P*=.01) showed a significantly larger improvement in cognitive function than the control group (Z=–1.19, *P*=.24). The reduction in physical frailty in the intervention group (Z=–1.73, *P*=.08) was similar to that in the control group (Z=–1.89, *P*=.06). Improvement in walking speed based on the Timed Up-and-Go test was moderate in the intervention group (Z=–0.16, *P*=.11) and greater in the control group (Z=–2.52, *P*=.01). The recruitment rate was acceptable (17/33, 52%). Both groups had a 100% attendance rate. The intervention group had a higher completion rate than the control group. Training was terminated for one participant (1/9, 11%) due to minimal VR sickness (Virtual Reality Sickness Questionnaire score=18.3/100). Two participants (2/8, 25%) in the control group withdrew due to moderate leg pain. No injuries were observed in either group.

**Conclusions:**

This study provides preliminary evidence that the VR simultaneous motor-cognitive training is effective at enhancing the cognitive function of older people with cognitive frailty. The effect size on frailty was close to reaching a level of significance and was similar to that observed in the control group. VR training is feasible and safe for older people with cognitive frailty.

**Trial Registration:**

ClinicalTrials.gov NCT04467216; https://clinicaltrials.gov/ct2/show/NCT04467216

## Introduction

### Background

Cognitive frailty refers to a clinical syndrome where physical frailty and mild cognitive impairment (MCI) coexist, and excludes concurrent dementia [1,2]. Cognitive frailty is associated with a higher risk of developing dementia, depression, malnutrition, and dependency [3,4], and is a common clinical syndrome among community-dwelling older people with a prevalence rate ranging from 4.4% to 19.9% [5-7]. Nevertheless, it is a reversible condition, particularly when treated at an earlier stage [8]. Therefore, cognitive frailty is regarded as a novel target for the prevention of elderly dependency and is a potential target for the secondary prevention of dementia [9,10].

Motor-cognitive training refers to a combination of physical exercise and cognitive training, and can be classified into two categories: (1) sequential motor-cognitive training (ie, motor and cognitive trainings are conducted separately) and (2) simultaneous motor-cognitive training (ie, motor and cognitive trainings are conducted concurrently) [11]. Evidence shows that physical exercise (eg, brisk walking) and cognitive training are two components of training that are effective at yielding clinical benefits for older people with cognitive frailty in terms of their cognitive function, physical frailty, and physical performance [12-14]. A systematic review showed that either simultaneous or sequential motor-cognitive training is more effective at promoting cognitive function than a single physical or single cognitive exercise [15].

The Guided Plasticity Facilitation framework postulates that simultaneous motor-cognitive training might lead to greater improvements in cognitive function through enhanced neuroplasticity [11]. The explanation for this is that when a task demands simultaneous cognitive and physical functioning, superadditive synergistic effects emerge from the facilitation effects of physical exercises and the guidance effects of cognitive exercises. As a result, synaptogenesis and neurogenesis could be fostered, leading to improved cognitive function. Studies have shown that motor-cognitive training is effective at improving physical performance (eg, walking speed), brain functional network as demonstrated by a resting-state functional magnetic resonance imaging (fMRI) scan, and cognitive function (eg, executive control, paired-associates learning) [16-18]. However, these studies compared the effects of motor-cognitive training with those of a passive control or single-component control (ie, either physical exercise or cognitive training). Clinical evidence showing that simultaneous motor-cognitive training is more effective than the sequential counterpart is lacking. Furthermore, no studies have been performed specifically on older people with cognitive frailty.

Virtual reality (VR) systems immerse users in a virtual environment by replacing the visual and aural environments to achieve a sense of presence so that users perceive themselves as being part of the virtual environment [19]. VR has been used in training because it is capable of simulating real-life scenarios in a controlled, safe, and ecologically valid setting [20]. A VR system can serve as a platform from which to launch cognitive and motor training programs. As a result, the effects of training are theoretically more easily translated in real-life environments. Moreover, adding gaming elements to the training to yield therapeutic effects (ie, serious games) could increase the motivation of participants to engage in the training [21]. A systematic review indicates that VR has been used for the rehabilitation of people with various neurological disorders (eg, stroke, cerebral palsy, spinal cord injuries), and that it is effective at improving the participants’ cognitive function, motor function, and community participation [22]. Another systematic review has shown that game-based VR interventions are potentially effective at improving the motor function and quality of life of people after stroke [23]. That said, various barriers to the use of VR in neurorehabilitation have also been reported, including the complex technical setup, simulation sickness, and the suitability of the design and its development for a population [24]. The generalizability of the training effects of the VR rehabilitation in different populations is unclear. Evidence is lacking on the effects and feasibility of a simultaneous motor-cognitive training program launched on a VR system for community-dwelling older people with cognitive frailty.

### Objectives

Based on this background, the aims of this study were to (1) explore the feasibility of VR simultaneous motor-cognitive training for older people with cognitive frailty; and (2) examine the effects of a VR simultaneous motor-cognitive training program compared with those of a non-VR sequential motor-cognitive training program in the community on the cognitive function, frailty, and physical function of older people with cognitive frailty.

## Methods

### Trial Design

This pilot study was designed as a single-blinded, single-centered, parallel-group randomized controlled trial (RCT). Therefore, we followed the reporting format of the CONSORT (Consolidated Standards of Reporting Trials) 2010 guideline [25]. The trial was registered with ClinicalTrials.gov (identifier number NCT0446726).

### Participants

#### Recruitment

The participants were recruited at an elderly community center in Hong Kong. The center provides social and recreational services for those 60 years of age or over. The staff of the center invited participants through a poster advertisement and telephone calls. Trained research assistants subsequently screened the potential participants according to the following eligibility criteria.

#### Inclusion Criteria

The inclusion criteria were (1) age ≥60 years; (2) community-dwelling, defined as living at home and not having stayed in a long-term care facility (eg, a nursing home) in the past 12 months; and (3) cognitive frailty, defined as the coexistence of MCI and physical frailty without being severe enough to have dementia. MCI was measured according to (1) a Montreal Cognitive Assessment (MoCA) score ≤25 [26] and a Clinical Dementia Rating of 0.5 [27]. Frailty status was measured on a scale from prefrail to frail, using the Fried Frailty Phenotype (FFP) scale, which assesses five components of frailty, namely handgrip strength, walking speed, physical activity level, exhaustion, and weight loss with an FFP score of ≥1 [28].

#### Exclusion Criteria

Participants were excluded if they had (1) a diagnosis of dementia, according to the subject’s medical record; (2) probable dementia, as defined by a MoCA score ≤18 [26]; or (3) restricted mobility, as defined by a Modified Functional Ambulatory Classification below Category 7 (ie, outdoor walker) [29]. This criterion was used because the subject might be unable to complete the motor-training exercises.

### Interventions

#### Design

There were two interventions employed in this study: the VR partially simultaneous motor-cognitive training (ie, the experimental group) and the non-VR sequential motor-cognitive training (ie, the control group). Both interventions were provided complementarily to eligible participants. There were no interventions that were provided in addition to the targeted intervention. However, the research team did not forbid participants from taking part in other usual activities that they had been participating in regularly. The staff at the elder center would call the participants to remind them to attend if they were not present on time. We rescheduled the intervention for participants no later than 1 week if they missed an appointment.

#### Experimental Group

The intervention provided many tasks taxing participants’ motor and cognitive functions simultaneously through a training system developed by the research team. In the training, some tasks demanded motor and cognitive functions simultaneously, while other tasks demanded cognitive functions only. This design did not demand all tasks to tax motor and cognitive function simultaneously, as shown in Table 1. This approach could better reflect reality because not all tasks in daily living performed by older people demand motor and cognitive functions simultaneously. Nevertheless, to ensure an adequate amount of motor-cognitive training, the majority of the tasks demanded motor-cognitive functions simultaneously (6/8, 75%). The training system included an immersive VR platform with a head-mounted VR display with a pair of headphones and wireless handheld controllers (HTC VIVE Focus Plus), under-desk ergometer with adjustable cycling resistance (DeskCycle 2), motion sensor, wrist-worn heart rate sensor (Polar OH1), and video game developed by the team (see Multimedia Appendix 1)

Cognitive training was delivered through a serious video game codeveloped by a team of health care academics specializing in the care of older people with cognitive impairment and in designing VR applications. The team engaged a technical company to produce the game. The video game included training in eight daily living tasks commonly performed by older people in Hong Kong. As shown in Table 1, these eight tasks were arranged in eight progressive stages. They included orientation, finding a bus stop, reporting lost items, finding a supermarket, grocery shopping, cooking, finding a travel hotspot, and bird watching. These tasks tax cognitive functions such as visuospatial (eg, wayfinding), calculation (eg, settling payment), memory (eg, recalling items while grocery shopping), reaction time (eg, flipping eggs when cooking), and attention (eg, getting off a bus). Each week featured tasks involving two levels of difficulty in terms of cognitive demands (eg, more distractors, a higher complexity of items to be memorized, a shorter time for reaction). If the participant could complete the lower level in the first session in the week, they could proceed to the higher level in the second session of the same week. Motor training was provided by cycling on an ergometer, which allows cycling resistance adjustments to be made to increase the effort of cycling. The training system requires the participants to travel in the virtual world of the game through cycling on the ergometer while simultaneously participating in the cognitively demanding daily-living tasks.

**Table 1:** Description of the contents of the intervention.

Week	Name	Description	Demanding functions
1	Orientation	Participants were instructed to learn all of the commands in the game (eg, movement control, item selection) through a standardized tutorial package	N/A^a^
2	Finding a bus stop	Participants were asked to find a bus stop on a given route in a city; visuospatial function and attention were required	SMC^b^
3	Reporting lost items	Participants were asked to report to police some items that were lost in the street; problem-solving and visuospatial function were required	SMC
4	Finding a supermarket	Participants were asked to find a particular supermarket in the city; visuospatial function and attention were required	SMC
5	Grocery shopping	Participants were asked to shop in a supermarket for a list of food items, which they were told at the beginning of the game; memory and attention were required	SMC
6	Cooking	Participants were asked to flip eggs at a specific time interval; mental processing speed was required	C^c^
7	Finding a travel hotspot	Participants were asked to find a travel hotspot in a park; visuospatial function and attention were required	SMC
8	Bird watching	Participants were given a list of birds and asked to identify those that they had been shown at the beginning of the game as being present in a park; attention and memory were required	SMC

^a^N/A: not applicable.

^b^SMC: simultaneous motor-cognitive training.

^c^C: cognitive training.

Tailoring of the training was allowed. The level of difficulty, cycling resistance, and target cycling distance could be adjusted according to the participant’s preference and previous cycling performance. The settings were determined at the beginning of each training session. Both the interventionist and participant mutually agreed on the settings before each session of training started.

A similar pilot RCT employed VR motor-cognitive training for older people with MCI used a dosage of two 30-minute sessions per week for a total of 6 weeks [30]. Improvements on cognitive function and walking speed were noted but the effects on cognitive function were small. To assure that an adequate intervention dose is delivered for the desired effect while balancing the tolerance of VR by older people, our pilot study increased the total intervention dose from 6 to 8 weeks while keeping the twice-weekly sessions at 30 minutes each. The intervention lasted for 8 weeks with 2 sessions per week. Each training session lasted for 30 minutes. One new stage was added per week. Participants started the training from stage 1 every time and passed the stages cumulatively. For example, they participated in stage 1 only in week 1; by week 3, they would have completed stages 1, 2, and 3. The aim of this design was to ensure that the participants had sufficient time to learn through repeated practice, while at the same time exploring new stages to sustain their motivation through a sense of fun.

The training sessions were held in an elderly community center. To complete the training, the participants mostly followed the auditory and written instructions provided by the VR system. A trained research assistant provided one-to-one standby assistance to the participants throughout the training period to solve any technical problems the participants might encounter.

#### Control Group

The intervention for the control group involved providing motor and cognitive training sequentially on a non-VR platform. Materials included a tablet computer (Microsoft Surface Pro 7) and an under-desk ergometer (DeskCycle 2). Cognitive training was provided by a series of cognitive games performed on a tablet computer. The cognitive games included (1) Card Pairs (ie, attention), (2) Mind Game Double Memory (ie, memory), (3) Flashcard Maths (ie, calculation), and (4) Mind Game Double Connect the dots (ie, visuospatial); see Multimedia Appendix 2. Participants were asked to cycle on the ergometer to complete the motor training. The four games were all planned by level of difficulty according to the demand on the cognitive load (eg, more distractors, a higher complexity of items to be memorized, a shorter time for reaction). The motor and cognitive training were provided sequentially (ie, cognitive training followed by motor training).

The intervention lasted for 8 weeks with 2 sessions per week. The dose was comparable to that in the intervention group. Each training session lasted for 30 minutes, which included tablet-based cognitive training for 15 minutes followed by motor training for 15 minutes. Two cognitive games were offered to the participants in each session. The participants continued the game levels from the previous session. During the cycling segment of the session, the participants were not allowed to do anything other than cycling (eg, watch TV, browse on their smartphone).

The training segments in the control group were also held in the elderly community center. The participants mostly followed the written instructions provided in the cognitive games. The participants were only provided with an ergometer and were encouraged to practice cycling at their preferred pace and level of resistance. A trained research assistant provided one-to-one standby assistance to the participants throughout the training period to solve any technical problems they might encounter.

### Outcomes

#### Main Variables of Interest

We collected two types of data: demographic and outcome data. Demographic data were collected at baseline (T0) and outcome data were collected at both baseline (T0) and the week after completion of the intervention (T1). The data were collected face to face by trained research assistants.

The demographic data included age, gender, BMI, marital status, level of education, and number of chronic illnesses as defined by a confirmed medical diagnosis documented in the participants’ medical records according to the diseases listed on the Charlson Comorbidity Index [31].

The outcome variables included global cognitive function, physical frailty level, walking speed, and feasibility.

#### Cognitive Function

Cognitive function was measured using the MoCA [26], which contains 30 dichotomous items. A correct answer for one item is accorded a score of 1 point. Total scores range from 0 to 30, with a higher score indicating better cognitive function. The test has been found to have good validity in detecting MCI (sensitivity=0.90, specificity=1.00) [26].

#### Frailty

Frailty was measured using the FFP [28], which quantifies the phenotypes of frailty according to five components (ie, weight loss, exhaustion, low physical activity, slow walking speed, and weakness) using physical performance tests and questionnaires following the Fried guideline. FFP scores range from 0 to 5, with 1 point assigned for the presence of one component. A higher FFP score indicates a higher frailty level. Scores of 0, 1-2, or 3-5 are respectively classified as robust, prefrail, or frail.

#### Walking Speed

Walking speed was measured by the Timed Up-and-Go (TUG) test [32], which quantifies the total time needed for participants to stand up, walk 3 meters, turn around, walk back to the chair, and sit down. Community-dwelling older people between 65 and 85 years of age are expected to be able to perform the TUG task within 12 seconds [33]. The TUG test has been reported to have moderate reliability in community-dwelling populations (intraclass correlation coefficient=0.56) [34] and has also been employed to identify slow walking speed in older people with frailty [35].

#### Feasibility

Feasibility was measured by adherence, adverse outcomes, and successful learning. Adherence was measured by the intervention attendance rate of completers (ie, those who did not withdraw from the study), the intervention completion rate (ie, the number of completers divided by the number of participants at baseline), as well as by the level of engagement in ergometer cycling (ie, the distance cycled and energy consumed in cycling as measured by the ergometer) over the intervention period. Adverse outcomes were measured using the Virtual Reality Sickness Questionnaire (VRSQ) [36] because simulator sickness is the most frequently reported adverse event in VR-based training [37]. The VRSQ consists of nine commonly observed simulator sickness symptoms, including general discomfort, fatigue, eye strain, difficulty focusing, headache, fullness of head, blurred vision, dizziness, and vertigo. The severity of each symptom is rated using a 4-point Likert scale (ie, 0=never, 3=very). The total score was computed by the summation of all item scores and was then converted to a percentage score. A higher score indicates higher severity in VR sickness. The VRSQ was validated as a reliable tool (Cronbach α=.847-.886) [36]. The participants of both groups were asked an open-ended question (ie, “What uncomfortable symptoms did you experience during and after the training?”) immediately after the intervention to identify other possible adverse outcomes. Successful learning was measured by the trend in completion time over time. A progressive reduction in completion time indicates successful learning, because it implies that the participants have learned to be more proficient in completing the cognitive tasks after repeated training.

### Sample Size

We did not estimate the sample size because we did not intend to test the effects for statistical significance. We planned to recruit a small sample of 15-20 participants for pilot testing.

### Randomization

A simple randomization method was adopted. A list of randomized numbers of either 1 or 0 (ie, 1=intervention group, 0=control group) was generated by an independent research assistant using Microsoft Excel. After subjects were screened for eligibility, a list of eligible participants was produced by the subject recruitment team. One author (LS) assigned the eligible participants to either the intervention or control group according to the list of randomized numbers. To ensure concealment, the list was kept by an independent research assistant who did not participate in the subject recruitment process. The randomized group allocation was performed after the data of all participants had been collected at baseline.

### Blinding

The outcome assessor was blinded to the group labels. However, it was not possible to blind the interventionists and participants in this study.

### Statistical Methods

The clinical profiles of the participants are described by the demographic data, reported according to the level of measurement. Continuous variables are reported as the median with IQR because of the small sample size. Categorical variables are reported as frequency and percentage. Differences in the demographic data of the groups were tested using either the Mann-Whitney *U* test or χ^2^ test according to the level of measurement.

For objective 1, we report the recruitment, attendance, and completion rates, and any adverse outcomes according to frequency and percentage. The VRSQ score in the intervention group is reported as the median and range.

For objective 2, we employed the Wilcoxon signed-rank test [38] to examine the within-group effects (ie, the difference in the outcomes observed between T0 and T1). We adopted this nonparametric test because the sample size was small. We also report the *Z*-score to represent the within-group effect size. The level of significance was judged at a threshold of .05. Intention-to-treat analysis was employed.

### Ethical Considerations

The study was approved by the Human Subjects Ethics Sub-Committee of The Hong Kong Polytechnic University (reference number HSEARS20200113003). Informed consent was obtained from all participants. To ensure safety, after the completion of every training session, the participants were required to sit at the elderly center for at least 10 minutes in case they were feeling any effects of VR sickness as assessed by the VRSQ (ie, any symptoms were rated as “3=very”) and that would affect their mobility. Otherwise, they would be sent to a clinic/hospital for medical treatment. If major injuries (eg, falls, severe VR sickness) occur, the study on the participant would be terminated. Participants were only allowed to leave if no adverse symptoms were reported. This study did not provide any forms of reimbursement to participants because this was considered to potentially confound the level of acceptance of the intervention.

## Results

### Participant Flow

**Figure 1:**
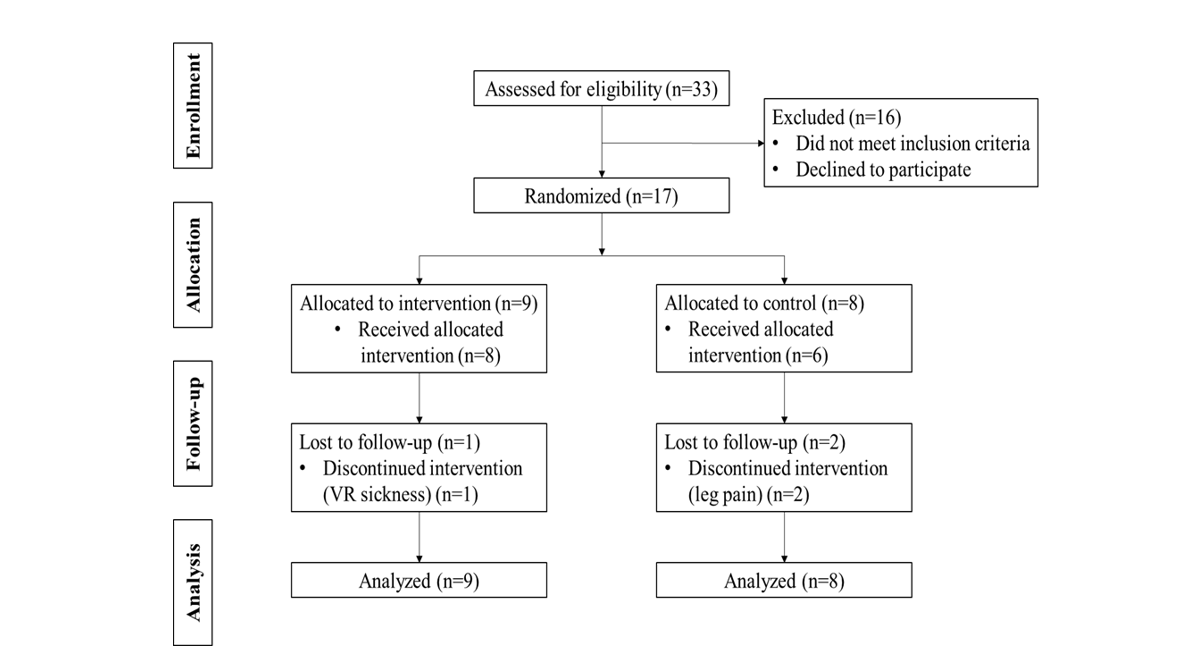
Participant flowchart. VR: virtual reality.

As shown in Figure 1, we assessed 33 subjects for eligibility and 16 were excluded because they did not meet the eligibility criteria (n=15) or did not consent to participate (n=1). The recruitment rate was acceptable (17/33, 52%). We randomly allocated 17 participants to the two groups (intervention group n=9, control group n=8). The research team terminated the training of 1 participant (11.1%) in the intervention group because they reported repeatedly experiencing mild VR sickness (VRSQ=18.3/100). Although the participant still wanted to continue with the training, the research team decided against this to ensure a high level of safety. In the control group, 6 participants completed the intervention. Two participants withdrew because they reported experiencing a moderate level of leg pain and were unable to participate in the cycling. All participants completed the outcome assessment at T1 and the data of all participants were employed in the data analysis. There were no missing data. The trial from recruitment to completion of follow-up was performed during the period from September to November 2020.

### Baseline Data

As shown in Table 2, most of the participants were female, widowed, had attained a primary level of education, had no VR experience, and had no chronic illnesses. The median age was 74 years, the median BMI was 22.9, the median MoCA score was 20.0, the median TUG time was 15.0 seconds, and the median grip strength was 14.0 kg. There were no significant differences between groups, except for the number of chronic illnesses (*P*=.04), with the participants in the control group reporting more chronic illnesses than those in the intervention group.

**Table 2:** Clinical profile at baseline.

Variables		All (N=17)	Intervention (n=9)	Control (n=8)	*P* value
**Gender, n (%)**					.93
	Male	2 (12)	1 (11)	1 (13)	
	Female	15 (88)	8 (89)	7 (88)	
Age (years), median (IQR)		74.0 (9.5)	73.0 (7.5)	77.5 (15.3)	.29
BMI, mean (SD)		22.9 (4.2)	24.4 (6.3)	22.2 (2.6)	.53
**Marital status, n (%)**					.46
	Single	0 (0)	0 (0)	0 (0)	
	Married	8 (47)	5 (56)	3 (38)	
	Widowed	9 (53)	4 (44)	5 (62)	
**Level of education, n (%)**					.48
	Secondary or above	5 (29)	2 (22)	3 (38)	
	Primary	9 (53)	6 (67)	3 (38)	
	No formal education	3 (18)	1 (11)	2 (25)	
**VR^a^ experience, n (%)**					N/A^b^
	Yes	0 (0)	0 (0)	0 (0)	
	No	17 (100)	9 (100)	8 (100)	
**Number of chronic illnesses, n (%)**					.04
	0	12 (71)	8 (89)	4 (50)	
	1-2	4 (24)	0 (0)	4 (50)	
	3 or above	1 (6)	1 (11)	0 (0)	
**Outcomes, median (IQR)**					
	Cognition: MoCA^c^	20.0 (4.0)	20.0 (4.0)	20.5 (4.5)	.62
	Walking speed: TUG^d^ (seconds)	15.0 (4.7)	14.0 (4.2)	15.5 (6.0)	.92
	Frailty: FFP^e^	2.0 (1.5)	2.0 (1.0)	2.0 (1.8)	.24
	Muscle strength: GS^f^ (kg)	14.0 (6.0)	14.7 (8.0)	14.0 (4.6)	.89

^a^VR: virtual reality.

^b^N/A: not applicable.

^c^MoCA: Montreal Cognitive Assessment.

^d^TUG: Timed Up-and-Go test.

^e^FFP: Fried Frailty Phenotype.

^f^GS: grip strength.

### Outcomes

#### Objective 1: Feasibility

**Figure 2:**
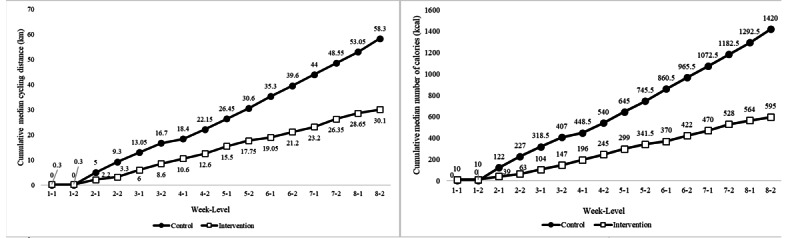
Comparison of the amount (left) and effort (right) of cycling exerted by the two groups over time.

With regard to adherence, the attendance rate for completers was 100% in both groups. The completion rate of the intervention group (8/9, 89%) was higher than that of the control group (6/8, 75%). As shown in Figure 2, the cycling distance of the control group (median 58.3 km, IQR 34.67) was greater than that of the intervention group (median 30.1 km, IQR 9.9), as measured by the ergometer. According to the ergometer, the cycling energy of the control group (median 1420 kcal, IQR 834) was higher than that of the intervention group (median 595 kcal, IQR 140). There was no significant difference between groups concerning total cycling distance (Z=–1.44, *P*=.15), whereas the difference in total cycling calories between the two groups was significant (Z=–1.93, *P*=.004).

The difference in cycling amount between the intervention and control groups was due to the difference in the design of the trainings. In the intervention group, participants only cycled as required by the tasks (eg, traveling in the virtual city for grocery shopping and finding a bus stop). When they participated in other tasks without movement (eg, cooking and getting off the bus at the right stop), simultaneous cycling was not needed. In the control group, cycling was unpaired with any other task. Most of the participants cycled continuously. Therefore, cycling-related energy expenditure between groups was significantly different. However, the difference in cycling distance between groups was not statistically significant. A factor that could account for the significant between-group difference in cycling energy expenditure was the participants’ individual adjustments to the cycling resistance. In a given cycling distance, the increase in cycling resistance requires a higher level of energy. As the cycling resistance of the control group surpassed that of the intervention group overall, their between-group difference in cycling-related energy expenditure became significant.

With regard to adverse outcomes, the vast majority of participants never experienced any symptoms of VR sickness. Overall, those who did feel such symptoms experienced mild symptoms, as detected by a median VRSQ score in the intervention group (n=9) of 4.63 (IQR 18.33). In the control group, two participants withdrew because they reported a moderate level of pain in the joints and muscles of their lower limbs. In the postintervention interview, they reported that the pain was exacerbated by the cycling, so that they were unable to continue the cycling training. No other symptoms causing discomfort were reported by the participants.

**Figure 3:**
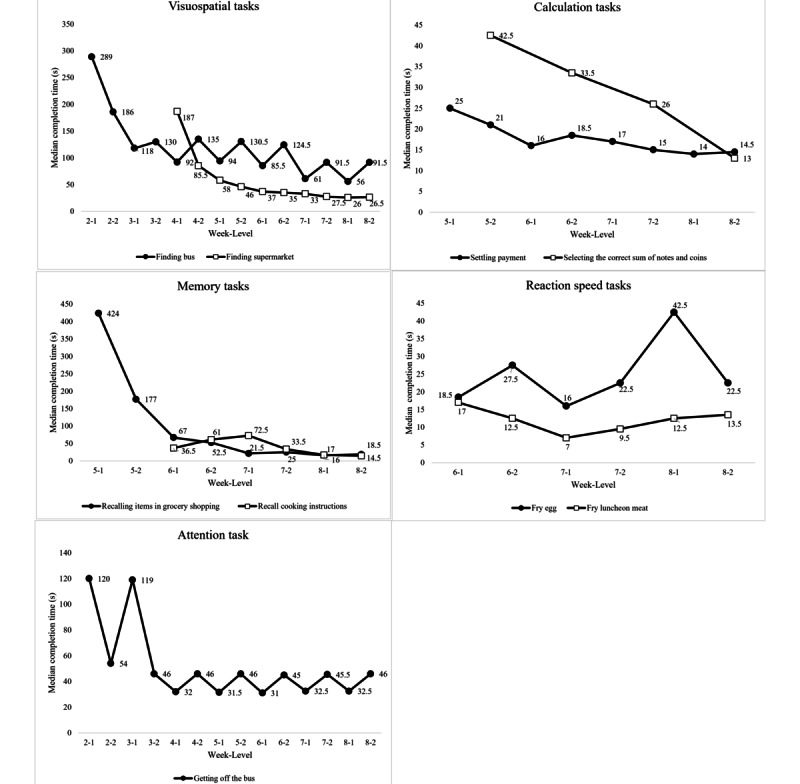
Amount of time taken to complete the cognitive tasks over the intervention period.

Successful learning among the participants was observed, as shown in Figure 3. During the intervention period, there was a gradual reduction in the time taken to complete all of the cognitive tasks as logged by the VR training system in week 2 (ie, visuospatial and attention tasks), week 4 (ie, visuospatial task), week 5 (ie, memory, attention, and calculation tasks), and week 6 (ie, reaction speed).

#### Objective 2: Intervention Effects

As shown in Table 3, the improvement in cognitive function in the intervention group was larger than that in the control group. The within-group effect of cognitive function was significant in the intervention group (*P*=.008) but not in the control group. After completion of the intervention, the reduction in frailty in the intervention group was similar to that in the control group. The within-group effects in both groups were close to reaching statistical significance. The improvement in walking speed as measured by the TUG test was greater in the control group than in the intervention group. The within-group effect was significant in the control group (*P*=.01) but not in the intervention group.

**Table 3:** Effects of the interventions by group.

Outcomes	Intervention (n=9)				Control (n=8)			
	Pre, median (IQR)	Post, median (IQR)	Z	*P* value	Pre, median (IQR)	Post, median (IQR)	Z	*P* value
Cognition: MoCA^a^	20.0 (3.5)	24.0 (5.0)	–2.67	.008	20.5 (4.5)	22.5 (4.5)	–1.19	.24
Walking speed: TUG^b^	14.0 (4.2)	10.5 (4.2)	–1.60	.11	15.5 (6.0)	10.8 (6.1)	–2.52	.01
Frailty: FFP^c^	2.0 (1.0)	1.0 (1.0)	–1.73	.08	2.0 (1.8)	1.0 (1.0)	–1.89	.06
Muscle strength: GS^d^	14.7 (8.0)	15.7 (4.7)	–1.24	.38	14.0 (4.6)	15.3 (5.4)	–1.35	.18

^a^MoCA: Montreal Cognitive Assessment.

^b^TUG: Timed Up-and-Go test.

^c^FFP: Fried Frailty Phenotype.

^d^GS: grip strength.

## Discussion

### Principal Findings

To the best of our knowledge, this is the first trial comparing the effects of a VR simultaneous motor-cognitive training program with those of a non-VR sequential motor-cognitive training program on older people with cognitive frailty. There are three key findings of this study. First, VR simultaneous motor-cognitive training is feasible (ie, good adherence and successful learning) and safe (ie, minimal adverse effects) for older people with cognitive frailty. Second, the preliminary findings of the VR simultaneous motor-cognitive training program suggest that it could be an effective method to enhance cognitive function in older people with cognitive frailty. Third, cycling-related energy expenditure is associated with greater improvement in walking speed, but could also lead to a higher dropout rate due to pain in the lower limbs.

A recent systematic review with a meta-analysis showed that older participants experienced a lower level of simulator sickness than younger participants when they used head-mounted displays for virtual application–based purposes [39]. However, the contents of the VR programs (eg, watching 360° videos, video game playing, and scenic viewing) in the studies varied greatly. The content of a program could play an important role in the severity of the simulator sickness symptoms experienced by participants. This study showed that VR simulator sickness was not a concern for many older people with cognitive frailty. Yet, for unknown reasons, some older people did experience prolonged VR sickness. To ensure safety, we recommend that potential participants first undergo a trial with VR; if VR sickness is observed and the severity is a concern, they should be excluded from participating in VR training. Even older people who did not experience any symptoms of VR sickness should be asked to stay behind for a short period after training. Only if they are observed to experience no symptoms of VR sickness in posttraining supervised walking should they be advised that it is safe to leave the community center.

Cognitive and exercise training stimulate similar neurobiological processes that produce a synergistic response through increasing cerebral blood flow, inducing angiogenesis in the cortex and cerebellum; however, the neurophysiological mechanism underlying the cognitive improvement is not fully understood [40]. This study provides preliminary evidence that simultaneous motor-cognitive training could produce a synergistic response on global cognitive function. This finding also concurs with the conclusion of a systematic review that simultaneous training was the most efficacious method for cognition, followed by sequential combinations [41]. Future studies should replicate this study with a tighter control (eg, a comparable training time, a comparable VR platform) and a larger sample size to examine if a synergistic response would indeed produce a stronger effect. Biomarkers (eg, fMRI) should also be added in future studies to confirm the underlying neurobiological mechanisms.

Walking speed is not only a marker of cognitive frailty but is also negatively associated with survival, physical function, and the risk of falls in older people [42-44]. An improvement in walking speed is an important modifiable health marker in older people. Physical activity promotes walking speed and, as a result, reduces the risk of disability and mortality in older people [2]. While physical activity could improve physical performance (eg, walking speed), it could also induce pain in older people, although the findings on this point in the literature are unclear. Physical activity beyond one’s level of physical endurance could cause exercise-induced pain through acute inflammation, whereas chronic pain could be alleviated by regular physical activity as explained by the exercise-induced analgesia model [45]. Older people with cognitive frailty might not regularly engage in physical activity or have low endurance. Should they suddenly increase the amount of their physical activity, they could experience exercise-induced pain or an exacerbation of their chronic pain. Although more physical activity could result in a significant improvement in walking speed, this study recommends that any increase in physical activity be steady to balance the risk of possible exercise-induced pain. The control group’s engagement in continuous cycling might have caused the participants to cycle more than usual but might have also led to more leg pain and a higher withdrawal rate from the study than might otherwise have been the case. It is possible that VR simultaneous motor-cognitive training with meaningful targets for increases in physical activity as directed by the cognitive games could minimize the risk of exercise-induced pain.

### Limitations

The limitations of this study are mostly related to its small sample size. First, the small sample size limited confidence in the effects that were observed. In particular, the effect sizes of the intervention on walking speed and frailty were close to statistical significance. Moreover, frailty is known to be associated with several chronic illnesses [46]. However, 70% of our sample reported no chronic illnesses. Caution should be exercised when generalizing findings to this population because this sample comprised older people with relatively fewer chronic illnesses in addition to MCI and frailty. Second, because of the small sample size, we used a nonparametric statistical test to examine only the within-group effect without testing the interaction effect between group and time. The cognitive improvement in the intervention group as measured by the MoCA could have been the result of repeated learning [47]. Third, although this study attempted to control many factors (eg, simultaneity), there were still many factors that could not be controlled to make the intervention and control groups more comparable. These factors included the cognitive training platforms (ie, 3D VR versus 2D tablet computer) and cognitive training time (ie, 15 minutes vs 30 minutes). Further studies should control for these factors more tightly to provide a stronger conclusion that simultaneous motor-cognitive training is more effective than sequential motor-cognitive training. Fourth, the study was only performed in one elderly center; thus, the mild symptoms of VR sickness observed in this group of older people should be interpreted with caution because this finding likely cannot be generalized to other settings.

### Conclusion

Preliminary evidence shows that VR simultaneous motor-cognitive training is effective at enhancing the cognitive function of older people with cognitive frailty. The effect size on frailty was close to reaching a level of statistical significance and was similar to that observed in the control group. The VR simultaneous motor-cognitive training program is feasible and can be safely applied to older people with cognitive frailty, although VR sickness was observed in a small number of participants. Future training sessions should exclude those who exhibit VR sickness at the eligibility screening phase and provide for adequate posttraining observations. Future studies should replicate this study by employing a larger sample so that its effects can be more confidently evaluated.

## Appendix

Multimedia Appendix 1 Setup of the VR motor-cognitive training.

Multimedia Appendix 2 Screenshots of the VR games used in intervention.

Multimedia Appendix 3 CONSORT-eHEALTH checklist (V 1.6.1).

## Abbreviations

FFP: Fried Frailty Phenotype
fMRI: functional magnetic resonance imaging
MCI: mild cognitive impairment
MoCA: Montreal Cognitive Assessment
RCT: randomized controlled trial
TUG: Timed Up-and-Go
VR: virtual reality
VRSQ: Virtual Reality Sickness Questionnaire
