# Supplementary figures and images for: Feasibility and Effects of Virtual Reality Motor-Cognitive Training in Community-Dwelling Older People With Cognitive Frailty: Pilot Randomized Controlled Trial
Source: JMIR Serious Games. 2021 Aug 6;9(3):e28400. doi: 10.2196/28400 (PMC8380584; doi:10.2196/28400)

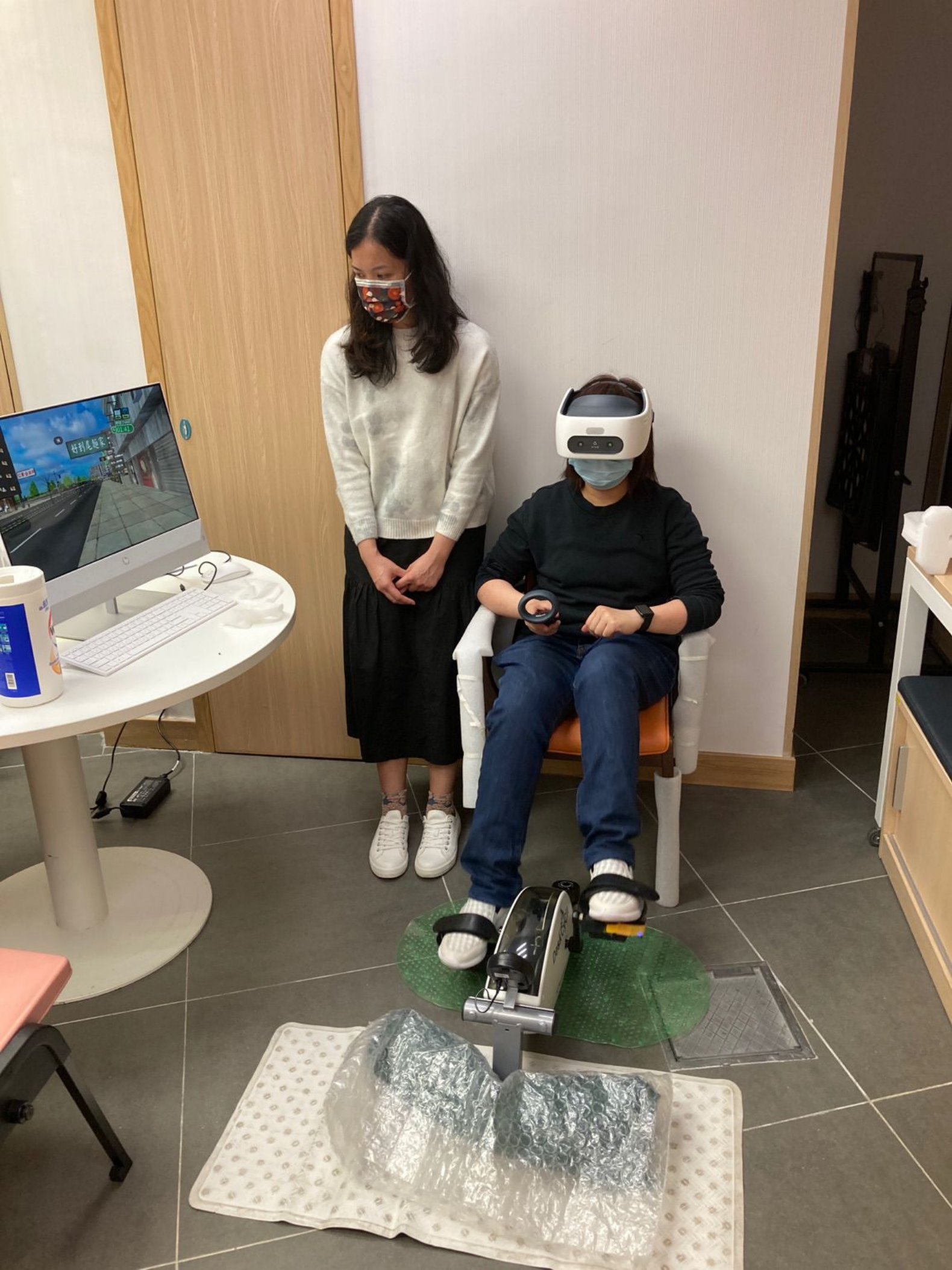

Supplement: Multimedia Appendix 1 [file games_v9i3e28400_app1.pdf]
