# Supplementary material for: Feasibility and Effects of Virtual Reality Motor-Cognitive Training in Community-Dwelling Older People With Cognitive Frailty: Pilot Randomized Controlled Trial
Source: JMIR Serious Games. 2021 Aug 6;9(3):e28400. doi: 10.2196/28400 (PMC8380584; doi:10.2196/28400)
Supplement: Multimedia Appendix 2 [file games_v9i3e28400_app2.pdf]

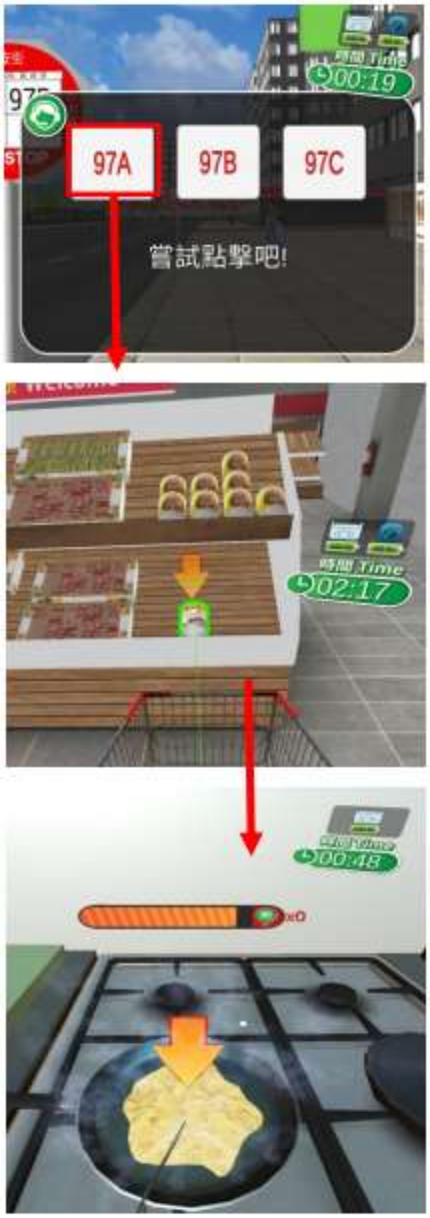

Week 1: Orientation

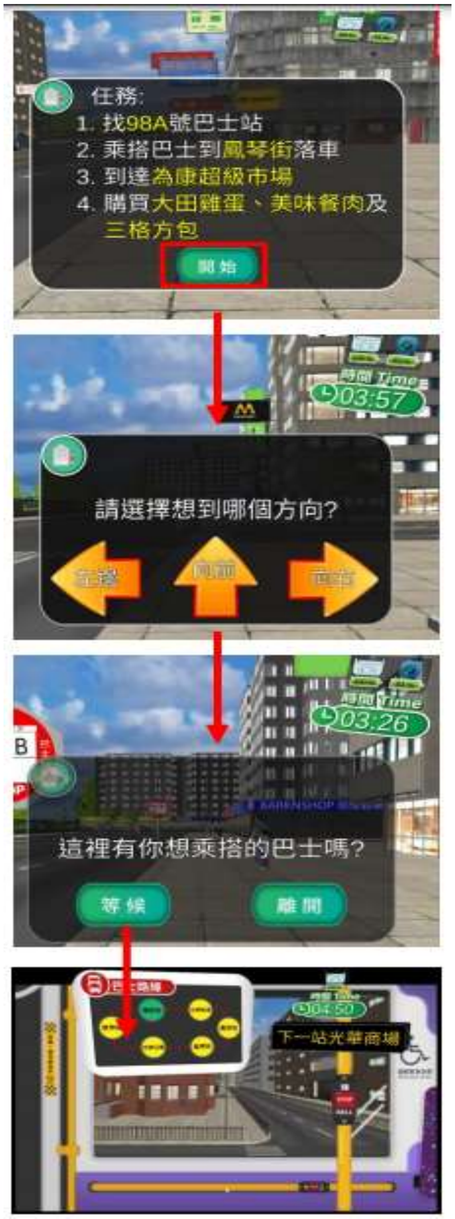

Week 2: Finding a bus stop

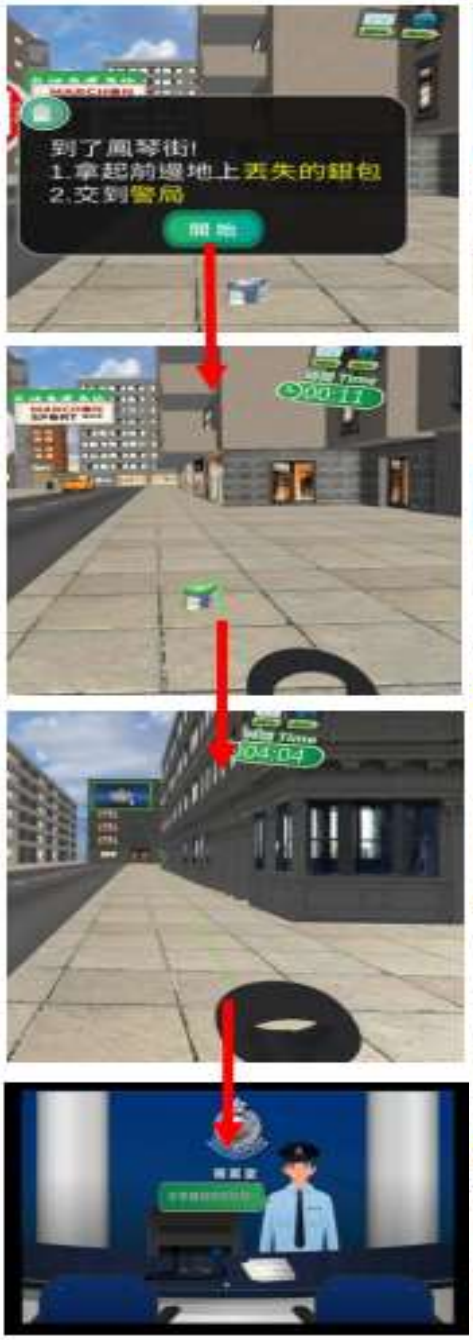

Week 3: Reporting lost items

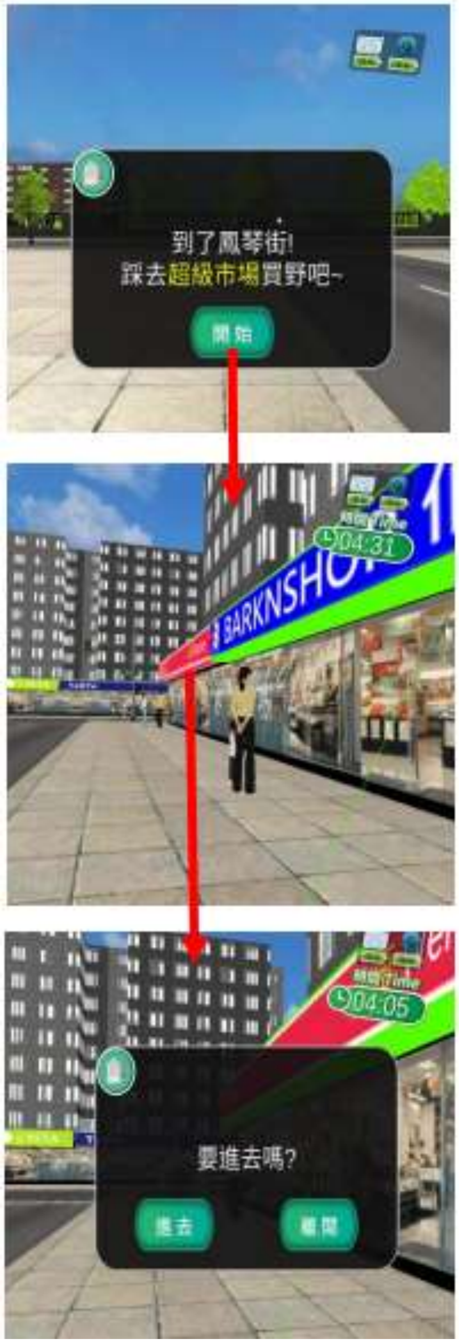

Week 4: Finding a supermarket

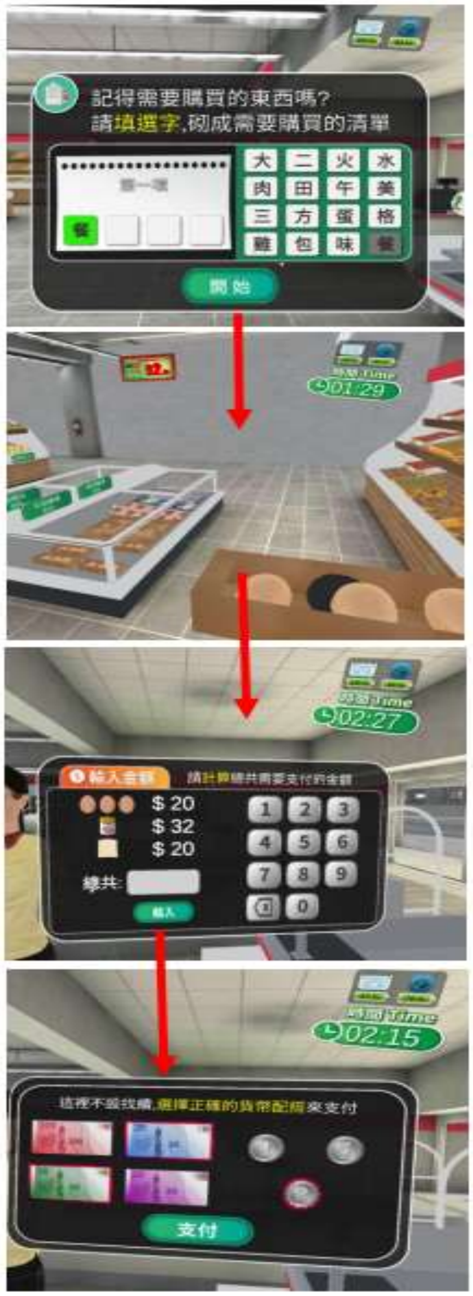

Week 5: Grocery shopping

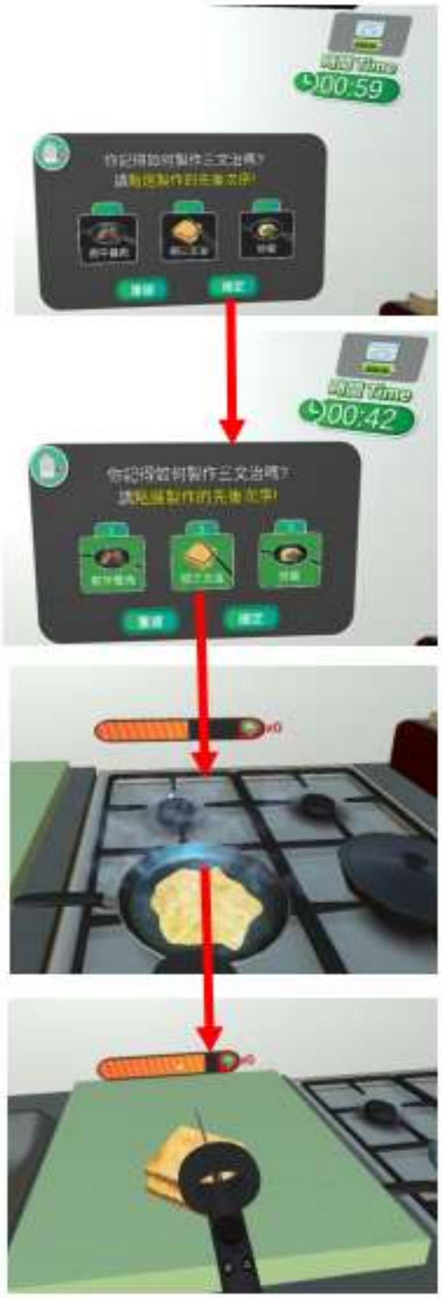

Week 6: Cooking

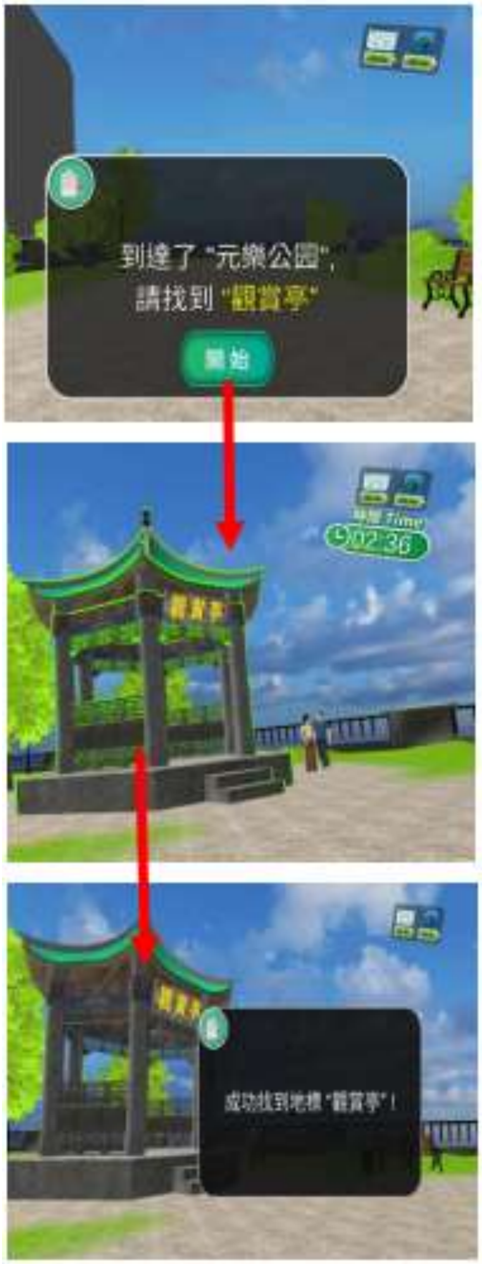

Week 7: Finding a travel hotspot

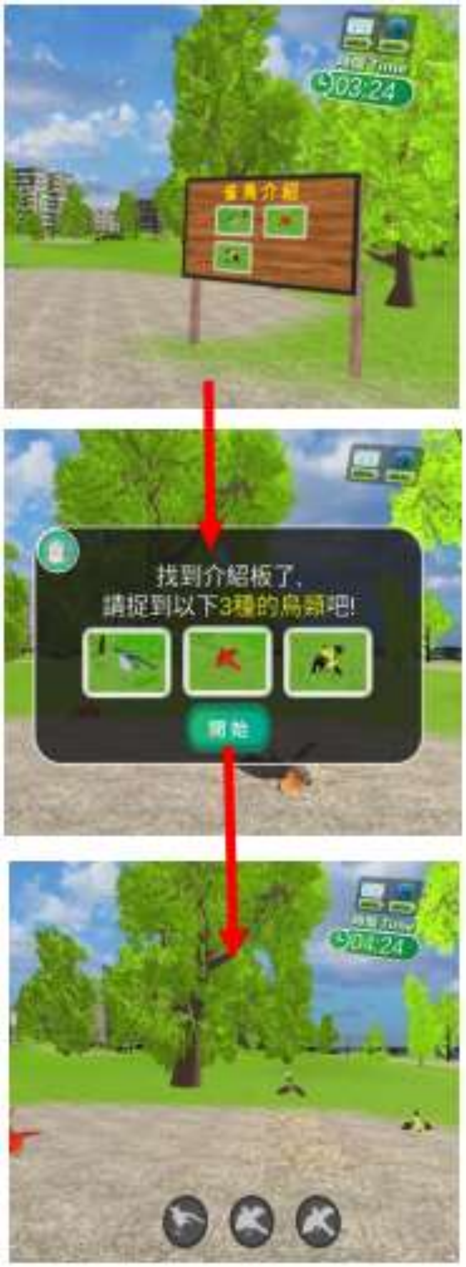

Week 8: Bird watching
